# Validation of a modified obstetric comorbidity index for prediction of postpartum adverse events including fetal morbidity - a retrospective cohort study from Qatar

**DOI:** 10.1186/s12884-024-06612-x

**Published:** 2024-06-08

**Authors:** Fouad Chaalan, Fathima Minisha, Zehra Zaidi, Alaa Babekar, Huda Abullah Hussain Saleh, Zeena Saeed Bu Shurbak, Mariam Al Baloushi, Alaa Alnaama, Husham Ahmed, Isaac Babarinsa, Nader Al Dewik, Abdulrouf Pallivalapila, Victor Olagundoye, Thomas Farrell

**Affiliations:** 1 Department of Obstetrics and Gynecology, Women’s Wellness and Research Centre, Hamad Medical Corporation, Doha, Qatar; 2 Women’s Wellness and Research Centre, Executive Director of Quality and Safety, Hamad Medical Corporation, Doha, Qatar; 3 Department of Research, Women’s Wellness and Research Centre, Hamad Medical Corporation, Doha, Qatar; 4 Chair of Research, Department of Research, Women’s Wellness and Research Centre, Hamad Medical Corporation, Doha, Qatar

**Keywords:** Obstetric comorbidity index, Severe maternal morbidity, OBCMI, Maternal risk assessment, Fetal morbidity

## Abstract

**Background:**

The Obstetric Comorbidity Index (OBCMI) is an internationally validated scoring system for maternal risk factors intended to reliably predict the occurrence of severe maternal morbidity (SMM). This retrospective cohort study applied the OBCMI to pregnant women in Qatar to validate its performance in predicting SMM and cumulative fetal morbidity.

**Methods:**

Data from 1000 women who delivered in July 2021 in a large tertiary centre was extracted from medical records. The OBCMI index included maternal demographics, pre-existing comorbidities, and various current pregnancy risk factors such as hypertension, including preeclampsia, intrauterine fetal death, prolonged rupture of membranes and unbooked pregnancies. SMM was based on the ACOG consensus definition, and the cumulative fetal morbidity (CFM) included fetal distress in labour, low APGAR and umbilical artery (UA) pH, admission to neonatal intensive care (NICU), and hypoxic-ischemic encephalopathy (HIE). A c-statistic or area under curve (AUC) was calculated to determine the ability of OBCMI to predict SMM and CFM.

**Results:**

The median OBCMI score for the cohort was 1 (interquartile range- 0 to 2); 50% of women scored 0, while 85% (*n* = 842) had a score ranging from 0 to 2. Ten women (1%) scored ≥ 7; the highest score was 10. The incidence of SMM was 13%. According to the modified scoring system, the mean OBCMI score in those who developed SMM was 2.18 (± 2.20) compared to a mean of 1.04 (± 1.40) in those who did not (median 1, IQR:1–3 versus median 0, IQR: 0–2; *p* < 0.001). The incidence of CFM was 11.3%. The incidence of low APGAR score, HIE and NICU admission was nearly 1 in 1000. Around 5% of the babies had fetal distress in labour and low UA pH. For every 1 unit increase in OBCMI score, the odds of SMM increased by 44% (OR 1.44 95% CI 1.30–1.59; *p* < 0.001; AUC 0.66), and CFM increased by 28% (OR 1.28 95% CI 1.15–1.42; *p* < 0.001; AUC 0.61). A cut-off score of 4 had a high specificity (> 90%); 1 in 4 and 1 in 6 women with OBCMI score ≥ 4 developed SMM and CFM, respectively.

**Conclusion:**

The OBCMI performed moderately well in predicting SMM in pregnant women of Qatar and can be effectively used as a risk assessment tool to red-flag high-risk cases so that appropriate and timely multidisciplinary care can be initiated to reduce SMM and maternal mortality. The index is also helpful in predicting fetal morbidity; however, further prospective studies are required to validate OBCMI for CFM.

## Introduction

Severe maternal morbidity (SMM) can be considered as unexpected maternal outcomes of labour and delivery, resulting in adverse maternal health consequences and possibly maternal death [1]. Risk factors for maternal morbidity have the potential to identify women at increased risk of developing SMM, enabling timely interventions to reduce the occurrence or severity of the SMM and, in turn, reduce maternal mortality. Over the past decades, there has been an upward trend in the prevalence of SMM worldwide, coinciding with the rising caesarean section rate and increasing maternal age [2]. More than 60% of maternal mortality and morbidity are due to preventable factors [3], highlighting the need for appropriate risk screening and early identification of high-risk maternal factors to flatten this upward trend in SMM.

Unpublished data from Qatar (2019) identified 108 intensive care admissions related to pregnancy complications over three years (2.18 per 1000 deliveries); obstetric hemorrhage and sepsis accounted for approximately 60% of admissions. Qatar Early Warning Systems (QEWS) enable early identification of patients with abnormal vital signs at risk of clinical deterioration [4], resulting in appropriate escalation of care and timely intervention. However, this system is based on a reactive approach to changes in vital signs and may lack specificity to identify deterioration in the pregnant population due to the maternal physiological changes in pregnancy [5]. Therefore, in addition to such systems, a proactive method of risk assessment focussing on maternal comorbidities becomes necessary to red-flag high-risk patients to reduce SMM [6]. Such a risk assessment will help incorporate a multidisciplinary approach and help with the early mobilisation of the appropriate level of clinical expertise for the care of these patients [7].

The Obstetric Comorbidity Index (OBCMI) was generated in 2013 by combining various maternal comorbidities using prediction modelling techniques, assigning weights to each comorbidity based on their impact on SMM [8]. The combined numerical score has a moderately good ability to predict SMM and has been validated with similar results in different populations [9–12]. Additional morbidities were included in a modified OBCMI score in 2018 that reported a rapid increase in the risk of SMM with the increase in OBCMI score, with the odds increasing by 55% for every unit increase in OBCMI [13]. This score was developed specifically for SMM and not evaluated for predicting fetal morbidity. Since maternal morbidity is associated with higher fetal morbidity, it is possible to utilise OBCMI to predict fetal morbidity as well.

The application of the OBCMI in a high-risk maternity tertiary referral unit in Qatar will enable the triaging of patients admitted for delivery and the modification of management pathways to optimize patient care within the delivery room. This retrospective observational study evaluates the performance of OBCMI in predicting severe maternal morbidity and fetal morbidity in the pregnant women of Qatar.

## Methods

### Study design and setting

A retrospective cohort study of 1000 women delivering at Women’s Wellness and Research Centre (WWRC) during July 2021 was performed. WWRC is the only tertiary hospital in Qatar, with a delivery rate of around 15,000–18,000 deliveries per annum. The study was approved by the Medical Research Centre, Hamad Medical Corporation (MRC-01-22-067), with a waiver of informed consent as only existing data from medical records was used for the analysis. The STROBE checklist was used to prepare this manuscript.

### Participants

All women delivering at more than 24 weeks gestational age (GA) in July 2021 (randomly chosen month) were considered for inclusion. There were 1,892 live births recorded in the State of Qatar in July 2021 [14]; nearly 70% delivered at WWRC. A random selection of 1000 women delivering at WWRC during the chosen month was included in this study. The selection was done by sorting the health card numbers from smallest to largest and choosing the first 1000 women; since there is no way to predict the sequence of health card numbers, this process resulted in a random selection. There were no other exclusion criteria. The study period was from admission to the hospital for delivery up to 72 h postpartum.

### Variables

All data was extracted directly from medical health records by well-trained data collectors. The maternal demographic variables included age in years (categorized into five categories from < 30 years to ≥ 45 years), nationality (Qatari and Non-Qatari), parity- defined as the number of prior births of more than 24 weeks GA (categorized into nulliparous, multiparous- parity 1–4, and grand multiparous- parity > 4), and maternal body mass index (BMI).

The OBCMI scores were calculated using maternal demographics, past medical history, and current pregnancy risk factors according to prediction models developed and validated by Bateman et al. in 2013 [8] and later modified by Easter et al. in 2019 [13]. The scores were assigned to predictors based on their coefficients in the models. The highest weights were given to severe preeclampsia/ eclampsia and congestive heart failure (5 points each), followed by congenital heart disease/ cardiac valvular disease and pulmonary hypertension (4 points each), chronic ischemic heart disease/ cardiac arrhythmia, sickle cell disease, bleeding disorder or coagulopathy (3 points each), multiple gestation, intrauterine fetal demise (IUFD), systemic lupus erythematosus or autoimmune disease, mild or unspecified preeclampsia, gestational hypertension, preexisting hypertension, placenta previa including suspected accrete spectrum and abruption, epilepsy, cerebrovascular accident, human immunodeficiency virus infection (HIV) or substance abuse (2 points each), chronic renal failure, previous uterine surgery, asthma, preexisting diabetes on insulin, or alcohol abuse (1 point each), as detailed in Appendix 1.

We have adopted the scoring system by Easter et al. as the Validated scoring system in this study. Additionally, we created a Modified scoring system including two more variables- spontaneous rupture of membranes more than 48 h (SROM) and pregnancies lacking adequate antenatal care (unbooked), due to their relevance in our setting. Each of these variables were assigned a score of 2 due to their importance in increasing maternal and fetal morbidity. A review of the variables scoring 2 and above in OBCMI was done, and based on clinical experience and joint consensus, the decision was taken to assign scores of 2 to these variables.

Maternal age was scored according to categories: age > 44 years getting a score of three, 40–44 years scored two and 35–39 years scored one. Similarly, maternal body mass index (BMI) was scored according to categories: BMI > 50, getting a score of 3, and BMI > 40, scoring 2. The women were scored for gestational hypertension only in the absence of preexisting hypertension and any form of preeclampsia. Similarly, they were scored only for the highest BMI category and only for one age category. The range of total scores possible was from 0 to 46. The OBCMI scores were calculated for these women based on the indicators present when they were admitted to the labor ward for delivery.

Apart from the variables used to calculate the OBCMI, other pregnancy and labour variables collected were the type of labour analgesia or anesthesia (epidural, spinal or general anesthesia), mode of delivery (vaginal versus cesarean), GA at birth in completed weeks, and fetal birthweight in grams measured immediately after birth.

The outcome variables included severe maternal morbidity (SMM) and cumulative fetal morbidity (CFM). The SMM was defined as any of the severe maternal morbidity indicators of maternal end-organ damage occurring during labour, delivery and in the immediate postpartum period (and up to 72 h after birth, whichever is earlier). These included postpartum hemorrhage- defined as obstetric hemorrhage at the time of delivery that might require blood transfusion and/or other life-saving measures, admission to high dependency unit, requiring close monitoring and invasive life-saving procedures, surgical complications including visceral and vessel injuries, sepsis, cardiac, renal, and neurological complications, complications of severe preeclampsia, anesthesia complications and maternal death. The SMM was defined based on the American College of Obstetrics and Gynecology (ACOG) 2016 consensus definition [1].

For the CFM, the fetal morbidity indicators included were fetal distress in labor requiring cesarean delivery, low APGAR score at 5 min (< 6), low umbilical artery pH- less than 7.2, hypoxic-ischemic encephalopathy (HIE) as diagnosed by the consulting neonatologist after investigations, and neonatal intensive care (NICU) admission for respiratory concerns or asphyxia. Babies with any of the above factors in the immediate postpartum period would be considered as having serious fetal morbidity.

### Statistical analysis

The continuous demographic variables were reported as mean ± standard deviation (SD) or median ± interquartile range (IQR) depending on the variable distribution (assessed by histograms and Shapiro Wilk test). Factor variables were presented as frequencies and percentages of the total. The OBCMI score for the entire cohort was reported as median ± IQR (ordinal variable). The proportions of women with each OBCMI score were plotted in a cumulative frequency graph.

The maternal and fetal morbidity indicators were reported as frequencies and percentages of the total. The median OBCMI scores in those with the outcome and those without were compared using the Wilcoxon rank-sum test. Logistic regression models calculated the increase in the odds of each indicator for every unit increase in OBCMI, reported as odds ratios (ORs) and 95% confidence intervals (CIs). Wald p-values were reported, and a p-value less than 0.05 was used as the cut-off probability for considering the null hypothesis as false.

Receptor-operative characteristic (ROC) analyses were used to determine the sensitivity and specificity of each OBCMI score if used as a cut-off to predict maternal and fetal morbidity. The sensitivity and 1-specificity values were plotted to obtain ROC curves, and the area under the curve (AUC) with 95% CIs was calculated to determine the ability of the OBCMI scores to discriminate between those with or without morbidity. An AUC of ≥ 0.65 was expected for maternal morbidity based on past publications.

To determine an OBCMI score cut-off, a combination of sensitivity, specificity and number needed to harm (NNH) was used. The morbidity risk difference (RD) between those scoring below the cut-off and those above the cut-off was calculated, and the NNH was equal to 100/%RD. Since we were more concerned about reducing the false negatives, we aimed for a high specificity (> 90%), and high negative predictive value (NPV > 90%). Risk ratios and 95% CIs were calculated using generalized linear models (binary family).

The above analysis was initially done in the previously validated scoring system and then repeated in our modified version, including additional comorbidities. The performance of the models were compared by comparing the ROC curves and AUCs. A p value less than 0.05 was considered as the cut-off for statistical significance. All statistical analysis was done using STATA 18.0 BE (College Station, TX: StataCorp, LP).

## Results

A total of 999 women were included in the analysis. The demographics of the cohort are shown in Table 1. The mean age was 31 years (± 5.5), the majority (40%) belonging to < 30 years age group and 6% above 40 years. One-third of the cohort were Qatari nationals, with a median parity of 2 (IQR 1 to 3, SD = 1.7). The mode of delivery was by cesarean in 36% of the sample, with the median GA at birth being 38 completed weeks (IQR 37–39).

**Table 1:** Maternal demographics, pregnancy risk factors and OBCMI scores

Maternal demographics Total *N*=999		*n*	%*N*
Maternal age (years) Mean ± SD		30.9 ± 5.5	
Maternal age	<30 years	395	39.7%
	30-34 years	354	35.6%
	*35-39 years	184	18.5%
	*40-44 years	58	5.8%
	*≥45 years	3	0.3%
Nationality	Qatari	303	30.3%
	Non-Qatari	696	69.7%
Parity (number of births >24 weeks gestation) Mean ± SD		2.1 ± 1.7	
Parity groups	Nulliparous (0)	181	18.2%
	Multiparous (1–4)	733	73.6%
	Grand multiparous (>4)	82	8.2%
*BMI >40 kg/m^2^		47	4.7%
*BMI >50 kg/m^2^		2	0.2%
*Previous caesarean section or myomectomy		272	27.2%
*Diabetic on insulin		49	4.9%
*Autoimmune, haematological, neurological, cardiovascular disease, asthma, and chronic renal disease		35	3.5%
Current pregnancy risk factors			
**Unbooked pregnancy		34	3.4%
*Multiple gestation		28	2.8%
*Preeclampsia/ gestational hypertension/ chronic hypertension		34	3.4%
*Preeclampsia with severe features/ eclampsia		14	1.4%
**Rupture of membranes >48 h		19	1.9%
*Placenta previa/ accreta/ suspected abruption		22	2.2%
*Intrauterine fetal death		4	0.4%
Labor analgesia/ anaesthesia	None	340	34.2%
	Epidural	371	37.3%
	Spinal	272	27.3%
	General anaesthesia	12	1.2%
Mode of delivery	Vaginal	641	64.2%
	Caesarean	358	35.8%
Gestational age at birth (completed weeks) Median (IQR)		38 (37-39)	
Estimated fetal birthweight in grams #; Mean ± SD		3113 ± 584	
Obstetric Comorbidity index (OBCMI) Median (IQR)		**1 (0-2)**	

* Used for the calculation of the OBCMI score, ** additionally added in the modified scoring; SD- standard deviation; IQR- interquartile range; #- only singleton birthweights used; cardiovascular disease includes congenital and ischemic heart disease and arrhythmia; haematological disease includes sickle cell, hemoglobinopathies, bleeding disorders, coagulopathy, and use of anticoagulation; neurological disease includes epilepsy, cerebrovascular accident, and neuromuscular disorders

Almost 5% of the cohort had a BMI > 40 kg/m^2^ or were diabetic on insulin. Nearly a third (27.2%) had a previous uterine scar (cesarean or myomectomy), and 3.5% had a prior medical comorbidity (autoimmune, hematological, neurological, cardiovascular, pulmonary, or renal disease). 3.4% of the women had hypertension in pregnancy, with an additional 1.4% having severe preeclampsia or eclampsia. Four pregnancies resulted in IUFD, and 2.2% had placental complications such as placenta previa or abruption. 3.4% of the pregnancies were unbooked, 2.8% were multiple gestation (twins/triplets), and nearly 2% had SROM for 48 h. None of the women in the cohort were HIV positive or had a history of substance or alcohol dependency.

**Fig. 1:**
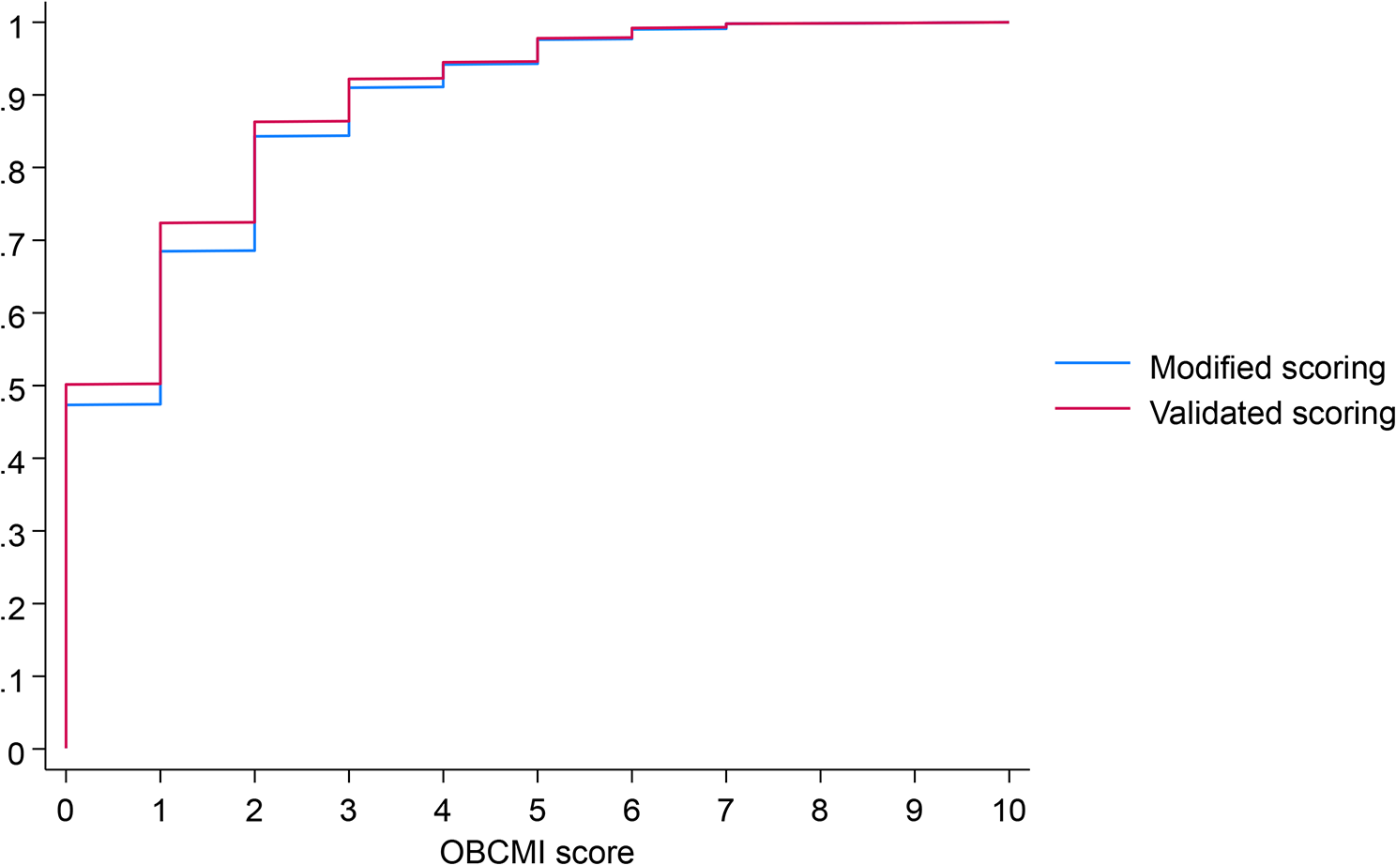
The cumulative frequencies of obstetric comorbidity index scores

The median OBCMI score, as per the original system, was 0 (IQR- 0 to 2). 50% of the women had a score of 0, while nearly 86% of women (*n* = 862) had a score ranging from 0 to 2. Only eight women (0.8%) had an OBCMI score ≥ 7. The highest score in this cohort was 10. Using our modified version, the mean score was 1(IQR 0 to 2), with 47% having a score of 0, 84% until score of 2, and ten women having high scores. Figure 1 shows the cumulative frequencies of both scoring systems.

SMM was observed in 130 women (13.1%), as shown in Table 2. As per the original system, the mean OBCMI in those who developed SMM was 2.09 (± 2.19) compared to a mean of 0.93 (± 1.33) in those who did not (median 1, IQR:0–3 versus median 0, IQR: 0–1; *p* < 0.001). As per our modified scoring, the mean score for SMM was 2.18 (± 2.20) compared to 1.04 (± 1.40) (median 1, IQR:1–3 versus median 0, IQR: 0–2; *p* < 0.001). For every 1 unit increase in OBCMI score, the odds of SMM increased by 44% (OR 1.44 95% CI 1.30–1.59; *p* < 0.001), which was similar to the increase in odds observed with the validated scoring.

**Table 2:** Maternal and fetal morbidity indicators during labor and delivery, including odds ratios for every unit increase in OBCMI score (95%CI)

Variable (*N*=999)	*n*	%*N*	Validated OBCMI		Modified OBCMI	
			OR for every unit increase in OBCMI (95%CI)	*p*-value	OR for every unit increase in OBCMI (95%CI)	*p*-value
Postpartum haemorrhage	42	4.2%	1.14 (0.96-1.36)	0.132	1.11 (0.94-1.32)	0.223
Admission to high dependency unit	55	5.5%	**1.96 (1.71-2.27)**	**<0.001**	**1.92 (1.67-2.21)**	**<0.001**
Surgical complications	42	4.2%	**1.28 (1.10-1.49)**	**0.002**	**1.27 (1.09-1.47)**	**0.002**
Sepsis	14	1.4%	1.25 (0.97-1.62)	0.087	1.25 (0.97-1.61)	0.081
Cardiac complications	6	0.6%	1.16 (0.75-1.78)	0.501	1.22 (0.82-1.81)	0.319
Chest complications	8	0.8%	1.31 (0.95-1.80)	0.096	**1.40 (1.04-1.88)**	**0.024**
Renal complications	21	2.1%	0.80 (0.46-1.39)	0.432	0.76 (0.44-1.33)	0.334
Neurological complications	11	1.1%	**1.48 (1.07-2.03)**	**0.017**	**1.44 (1.04-2.00)**	**0.029**
Anaesthesia complications	5	0.5%	0.74 (0.31-1.74)	0.486	0.92 (0.49-1.71)	0.792
Cumulative maternal morbidity (yes)	**130**	**13.1%**	**1.46 (1.33-1.63)**	**<0.001**	**1.44 (1.30-1.59)**	**<0.001**
Fetal distress in labor requiring caesarean	56	5.6%	**1.33 (1.17-1.52)**	**<0.001**	**1.36 (1.19-1.55)**	**<0.001**
Low APGAR score at 5 min (<6)	9	0.9%	1.28 (0.94-1.74)	0.118	**1.42 (1.08-1.87)**	**0.013**
Low umbilical artery pH (<7.2)	49	4.9%	1.08 (0.91-1.28)	0.384	1.08 (0.92-1.28)	0.357
Hypoxic Ischemic Encephalopathy	9	0.9%	0.91 (0.56-1.49)	0.706	0.97 (0.63-1.50)	0.887
NICU admission for asphyxia	12	1.2%	1.16 (0.86-1.57)	0.340	1.22 (0.92-1.62)	0.158
Cumulative fetal morbidity (yes)	**113**	**11.3%**	**1.25 (1.12-1.39)**	**<0.001**	**1.28 (1.15-1.42)**	**<0.001**

OR- odds ratios; CI- confidence interval; OBCMI- Obstetric comorbidity index; *p*<0.05 considered strong evidence against the null hypothesis. Odd ratios determined using logistic regression models with OBCMI as the independent variable

Statistically significant indicators contributing to SMM as per our modified scoring included admission to HDU, surgical, neurological and chest complications, the odds increasing by 92%, 27%, 40% and 44%, respectively, for every unit increase in OBCMI score (OR 1.92 95% CI 1.67–2.21, *p* < 0.001; OR 1.27 95% CI 1.09–1.47, *p* = 0.002; OR 1.40 95% CI 1.04–1.88, *p* = 0.024; OR 1.44 (1.04-2.00, *p* = 0.029 respectively), which was roughly similar to the validated scoring. The odds of sepsis and cardiac complications increased by 20% for every unit increase in OBCMI scores; however, they were not statistically significant. There were no maternal deaths in this sample.

The CFM was observed in 113 babies (11.3%). For the validated scoring, the mean OBCMI score in those with CFM was 1.64 (± 1.90) compared to a mean of 1.00 (± 1.45) in those who did not (median 1, IQR:0–2 versus median 0, IQR: 0–2; *p* < 0.001). For our modified system, the mean OBCMI score for CFM was 1.85 (± 2.02) compared to 1.10 (± 1.50) (median 1, IQR:0–3 versus median 1, IQR: 0–2; *p* < 0.001). The odds of CFM increased by 28% for every unit increase in OBCMI scores, as shown in Table 2, similar to the validated scoring. The significant contributors toward CFM included fetal distress in labour requiring CD and low APGAR score at 5 min (OR 1.36, 95% CI 1.19–1.55, *p* < 0.001; OR 1.42, 95% CI 1.08–1.87, *p* = 0.013, respectively). The low APGAR score was statistically significant with our modified scoring system but not so with the validated system.

**Fig. 2:**
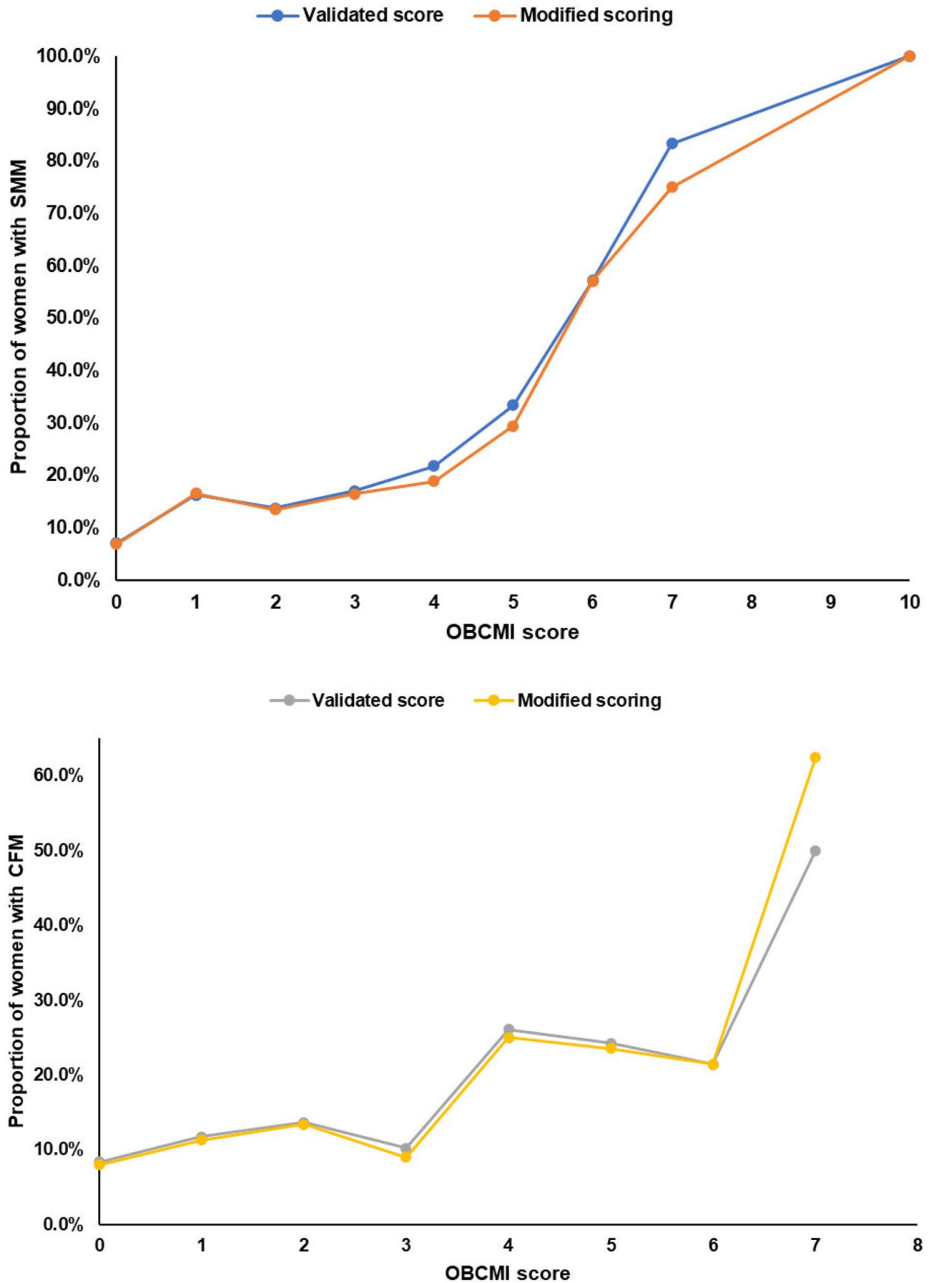
Proportion of women with maternal and fetal outcomes for each OBCMI score (according to the Validated scoring system and the Modified scoring system in this study)

**Fig. 3:**
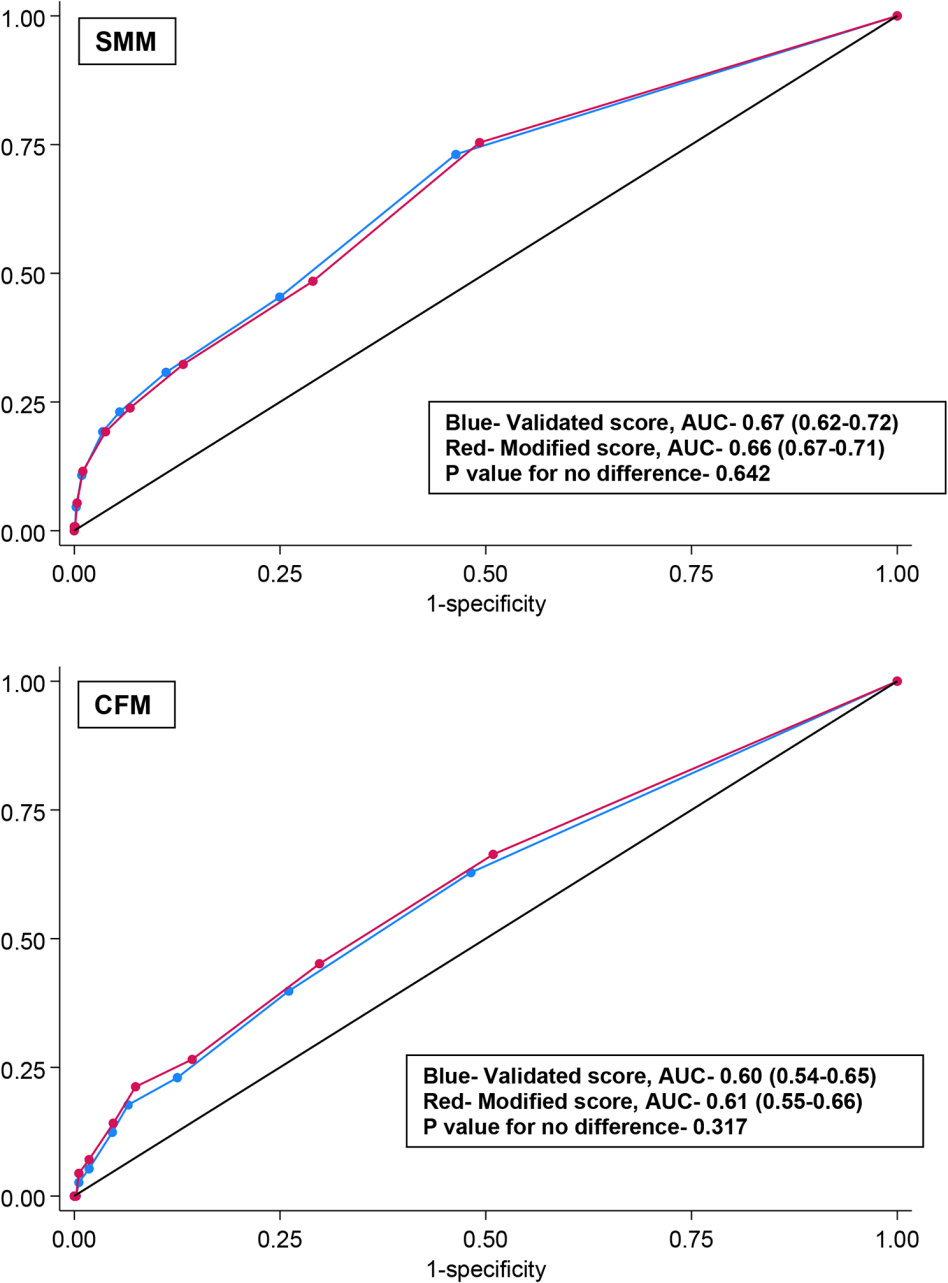
ROC curves for the prediction of severe maternal morbidity (SMM) and cumulative fetal morbidity (CFM) using Obstetric comorbidity score (OBCMI)- validated and modified

The proportion of women having SMM increased steadily as the OBCMI score increased; 75% of women with a score of 7 and above developed SMM, compared to only 7% with a score of 0 using both scoring systems (Fig. 2). The ROC analysis is shown in Fig. 3, with AUC being 0.66 (CI 0.61–0.71), showing moderate discrimination. There is no statistically significant difference between the AUCs of the validated and modified scoring systems (*p* = 0.642). The sensitivity and specificity for each OBCMI cut-off are shown in Table 3. Adopting a cut-off score ≥ 4 will give a specificity of > 90% and sensitivity of 23–24% for identifying SMM, with correct classification in 84–85% of cases and a NPV of nearly 90%, using either of the scoring systems. As per the validated scoring system, 38.5% of women with an OBCMI score ≥ 4 developed SMM compared to 10.9% of the remaining (Table 4); the absolute risk difference was 27.6% (Risk ratio 3.5, 95% CI 2.5-5.0). The NNH was calculated as 3.6 (absolute number 4); this meant that 1 in every four women with a score ≥ 4 had a risk of developing SMM. Similarly, according to the modified scoring system, the absolute risk difference for cut off ≥ 4 was 23.6% (Risk ratio 3.2, 95% CI 2.3–4.4), giving a similar NNH of 4.

**Table 3:** Sensitivity and specificity of each OBCMI score for predicting maternal and fetal morbidity

Validated OBCMI				Modified OBCMI				
Severe Maternal Morbidity				Severe Maternal Morbidity				
OBCMI cut-offs	Sensitivity	Specificity	Correctly classified	OBCMI cut-offs	Sensitivity	Specificity	Correctly classified	
≥0	100%	0.0%	13.0%	≥0	100%	0%	13.0%	
≥1	73.1%	53.6%	56.2%	≥1	75.4%	50.8%	54.0%	
≥2	45.4%	75.0%	71.2%	≥2	48.5%	71.1%	68.2%	
≥3	30.8%	88.8%	81.3%	≥3	32.3%	86.8%	79.7%	
≥4	23.1%	94.5%	85.2%	≥4	23.9%	93.2%	84.2%	
≥5	19.2%	96.6%	86.5%	≥5	19.2%	96.2%	86.2%	
≥6	10.8%	99.1%	87.6%	≥6	11.5%	99.0%	87.6%	
≥7	4.6%	99.8%	87.4%	≥7	5.4%	99.7%	87.4%	
≥9	0.8%	99.9%	87.0%	≥9	0.8%	99.9%	87.0%	
≥10	0.8%	100%	87.1%	≥10	0.8%	100%	87.1%	
**Cumulative fetal morbidity**				**Cumulative fetal morbidity**				
**OBCMI** **cut-offs**	**Sensitivity**	**Specificity**	**Correctly classified**	**OBCMI** **cut-offs**	**Sensitivity**	**Specificity**	**Correctly classified**	
≥0	100%	0.0%	11.3%	≥0	100%	0%	11.3%	
≥1	62.8%	51.8%	53.1%	≥1	66.4%	49.1%	51.1%	
≥2	39.8%	73.9%	70.1%	≥2	45.1%	70.3%	67.5%	
≥3	23.0%	87.5%	80.2%	≥3	26.6%	85.7%	79.0%	
≥4	17.7%	93.5%	84.9%	≥4	21.2%	92.6%	84.5%	
≥5	12.4%	95.4%	86.0%	≥5	14.2%	95.3%	86.1%	
≥6	5.3%	98.2%	87.7%	≥6	7.1%	98.2%	87.9%	
≥7	2.7%	99.4%	88.5%	≥7	4.4%	99.4%	88.7%	
≥9	0.0%	99.8%	88.5%	≥9	0%	99.8%	88.5%	
≥10	0.0%	99.9%	88.6%	≥10	0%	99.9%	88.6%	

OBCMI- Obstetric comorbidity index; Cut-off scores of four has specificity >90% and a negative predict value >90%

**Table 4:** Number needed to harm for OBCMI cut-off scores of 3, 4 and 5 (validated and modified OBCMI)

Validated OBCMI					Modified OBCMI					
OBCMI cut-offs Total *N*=999	Severe Maternal morbidity, *n*(%*N*)		Cumulative Fetal morbidity *n*(%*N*)		OBCMI cut-offs Total *N*=999	Severe Maternal morbidity, *n*(%*N*)		Cumulative Fetal morbidity *n*(%*N*)		
OBCMI score <3 *N*= 862	90 (10.4%)	RD =18.8% RR 2.8, *p*<0.001 **NNH= 5.3**	87 (10.1%)	RD =8.9% RR 1.9, *p*=0.002 **NNH= 11.2**	**OBCMI score <3** *N*= 842	88 (10.5%)	RD =16.3% RR 2.6, *p*=0.001 **NNH= 6.1**	83 (9.9%)	RD =9.3% RR 1.9, *p*=0.001 **NNH= 10.8**	
OBCMI score ≥3 *N*= 137	40 (29.2%)		26 (19.0%)		**OBCMI score ≥3** *N*= 157	42 (26.8%)		30 (19.1%)		
OBCMI score <4 *N*= 921	100 (10.9%)	RD =27.6% **RR 3.5,** ***p*** **<0.001** **NNH= 3.6**	93 (10.1%)	RD =15.5% **RR 2.5,** ***p*** **<0.001** **NNH= 6.5**	**OBCMI score <4** *N*= 909	99 (10.9%)	RD =23.6% **RR 3.2,** ***p*** **<0.001** **NNH= 4.2**	89 (9.8%)	RD =16.9% **RR 2.7,** ***p*** **<0.001** **NNH= 5.9**	
OBCMI score ≥4 *N*= 78	30 (38.5%)		20 (25.6%)		**OBCMI score ≥4** *N*= 90	31 (34.4%)		24 (26.7%)		
OBCMI score <5 *N*= 944	105 (11.1%)	RD =34.4% RR 4.1, *p*<0.001 **NNH= 2.9**	99 (10.5%)	RD=15.0% RR 2.4, *p*<0.001 **NNH= 6.7**	**OBCMI score <5** *N*= 941	105 (11.1%)	RD =31.9% RR 3.9, *p*<0.001 **NNH= 3.1**	97 (10.3%)	RD =17.3% RR 2.7, *p*<0.001 **NNH= 5.8**	
OBCMI score ≥5 *N*= 55	25 (45.5%)		14 (25.5%)		**OBCMI score ≥5** *N*= 58	25 (43.1%)		16 (27.6%)		

RD= Risk difference between groups; RR= Risk Ratio; NNH= Number needed to harm, NNH= 100/RD%; OBCMI- Obstetric comorbidity index

The rise in the proportion of babies with CFM was mainly seen for OBCMI scores ≥ 7. In the validate scoring system, 50% of women with scores of 7 developed CFM increasing to 63% when using our modified scoring (Fig. 2). The ROC analysis is shown in Fig. 3, the AUC being 0.61 (CI 0.55–0.66), showing low to moderate discrimination (no statistical difference between validated and modified scoring, *p* = 0.317). Similar to SMM, a cut-off score of ≥ 4 gave a specificity of > 90% and correct classification in nearly 85%, using either scoring systems. The modified scoring adopted in our study had a better sensitivity compared to the validated system (21% vs. 18%) for detecting CFM (Table 3). As per the validated system, 26.7% of women with an OBCMI score ≥ 4 developed CFM compared to 9.8% of the remaining (Table 4); the absolute risk difference was 17% (Risk ratio 2.5, 95% CI 1.7–3.9). This meant that 1 in every six babies born to women with an OBCMI score ≥ 4 had a risk of developing CFM. Similarly, for the modified scoring system, the absolute risk difference was 16.9% resulting in a NNH of 6 (Risk ratio 2.7, 95% CI 1.8-4.0).

A post-hoc power analysis was done to check the adequacy of the sample size. For SMM, the sample had more than 99% power to detect differences between those who had OBCMI score 0 versus score ≥ 1, as well as score < 4 and ≥ 4. For CFM, the power was 85% to detect differences between score 0 and ≥ 1; this increased to more than 95% for score < 4 versus ≥ 4.

## Discussion

This study demonstrates that screening pregnant women in Qatar with the obstetric comorbidity index at the time of elective or emergency admissions for delivery can be beneficial in predicting intrapartum and postpartum severe maternal morbidity. According to the modified scoring system used in our study, the odds of any maternal morbidity increase by 44% with every unit increase in OBCMI. Additionally, a cut-off score of 4 has a high specificity in predicting morbidity, with those scoring four and above having at least three times higher risk of SMM and an NNH of four. OBCMI has a moderate predictive ability when it comes to maternal morbidity. However, in the case of fetal morbidity, the discriminatory ability of OBCMI is lower. A score of 4 and above has a high specificity for fetal morbidity, but only 1 in 6 women with that cut-off score will develop CFM. The modified scoring was similar to the validated system for SMM but did better for predicting CFM.

The OBCMI was initially generated and validated by Bateman et al. in 2013 [8] in a sample of 854,823 completed pregnancies identified from Medicaid data from the US from 2000 to 2007. The model was generated using 21 maternal factors, and weights applied to each covariate depending on the magnitude of their coefficients for predicting SMM. They validated the score in 1/3rd of the cohort and compared them to existing indices, such as the Charlson/ Romano index [15, 16] and the Elixhauser index [17], that are used to combine morbidities into meaningful indices capable of predicting adverse events but not validated in the obstetric population. The study reported that OBCMI performed better than the existing indices for predicting SMM in an obstetric population.

Bateman et al. reported SMM in 1.16% of pregnancies in their sample. In our study, we report a 13% observed risk of SMM. This difference could be due to several factors. Bateman et al. developed the index on a vast general population using administrative data, whereas WWRC is the only tertiary referral unit in Qatar and is responsible for the care of all high-risk pregnancies in the country; therefore, the rate of SMM reported from WWRC is likely to be greater than in the general maternity population. Additionally, we included factors described by ACOG as not fulfilling criteria for SMM when they occur in isolation [1]. There is also a lower threshold for admission to the high-dependency unit in this facility due to the high-risk nature of the pregnancies. Additionally, the rates of maternal morbidity have increased by more than 50% over the past decades since the development of OBCMI [2], which could further explain our increased numbers.

The OBCMI was tested in a sample of 6000 women in Canada by Metcalfe et al. [12] using retrospective administrative data from 2007 to 08. More than 80% of the study population had an OCBMI score ≤ 2, similar to our results. They also report a 1.7% risk of the outcome, similar to the original study, and an AUC of 0.67 when using hospitalization data, which is similar to the results from our study. Various studies from different parts of the US during this period applied the Bateman OBCMI index to their populations over the past decade and reported a similar moderate predictive performance for similar rates of SMM [9–11].

One such example was a sizeable Californian study (> 3 million) from 2011 to 2017 to evaluate the performance of the index in different ethnic and socioeconomic groups [11]. The AUC of the ROC curves ranged from 0.68 to 0.76, and rates of SMM and OBCMI-adjusted rate differences varied based on ethnicities and factors of socioeconomic status like health insurance and education. Qatar has a multiethnic population of expatriates from over 90 countries. Since ethnicity or nationality is not part of the index, the differences observed between previous models and our study could also be explained by the population demographics in Qatar.

Prospective clinical validation was done in 2018, where OBCMI scores were calculated upon presentation to the hospital for delivery, and the women were followed up for maternal morbidity [13]. This study included four additional factors in calculating OBCMI (Appendix 1), which we adopted in this study. However, they report a 2% risk of SMM and a 1.53% increase in odds of morbidity for every unit increase in OBCMI, with a c-statistic or AUC of 0.8. The difference from our study might arise from the differences in the definition and measurement of individual maternal morbidity indicators. Additionally, we have included two more factors for calculating OBCMI in our study that are relevant to our setting. Many high-risk pregnancies receive initial ad hoc antenatal care in the private setting or abroad. Some receive no medical care prior to attending the hospital for delivery and are considered unbooked in WWRC, which increases their risk for SMM as antenatal preventive measures would be lacking. However, this would be relevant only for high-risk unbooked pregnancies and could explain the difference in the predictive ability of our modified OBCMI compared to the validated existing index.

The updated scoring of 2018 was applied in a population-based study on more than nine million women who delivered over 17 years in California [18]. The investigators used risk ratios for each comorbidity in the index to re-test and validate the weights or points given to each factor included in OBCMI. They reported that even though OBCMI performed well in identifying SMM, pre-existing hypertension, chronic renal disease and cardiovascular disease were underweighted in their sample, and maternal age and BMI were overweighted. They recommended including ethnicity and social factors in the index, as evidenced previously [11].

Similarly, the score was validated in a population in India, including women admitted for delivery over six months in 2019 [19]. They used the same SMM definition as the prospective study and reported 2.02 times increase in odds for every unit increase in OBCMI, with an AUC of 0.84. More than 80% of their population had a score ≤ 2 with a maximum OBCMI score of 10.

To the best of our knowledge, this is the first study to examine the association between OBCMI scores and fetal cumulative morbidity, although the performance was low-moderate. A good proportion of fetal morbidity (especially factors such as low Apgar score and low fetal scalp pH) occurs in mothers without significant comorbidities due to intrapartum factors independent of maternal risk factors. Hence, it’s reasonable to assume that OBCMI scores are less valuable in predicting fetal morbidity. Further prospective studies are required to refine the definition of fetal morbidity and validate the performance of OBCMI in predicting it. The modified system reported in our study performed better at predicting CFM because both additional variables have a statistically significant association with CFM. However, adding these variables did not affect the ability of OBCMI to predict SMM. Additionally, the sample size used was well powered to detect the differences we hoped to detect.

Currently, there is no scoring system in practice at WWRC for maternal comorbidities that can effectively predict SMM. Generating OBCMI scores upon admission would enable physicians to red-flag patients with higher scores so that they can be reviewed promptly by senior multidisciplinary teams, including obstetrics, anesthesia, intensivists, and neonatologists. The exploration of possible cut-off scores was done to ensure consistent clinical practice and has not been done previously. A score of 4 has very high specificity and NPV in predicting SMM and could accurately rule out women less likely to develop SMM. Steps are currently underway to incorporate OBCMI into the obstetric clinical practise in Qatar. Future audits of the scoring system after implementation can further evaluate the usefulness of OBCMI and the cut-off score in our population.

The large-scale studies done previously used hospital registries, linked datasets, and administrative data and extracted information using the ICD 9/10 coding systems, whereas we extracted data from patient health records. There is a possibility that these studies have underestimated the rates of SMM due to data collection methods. Conversely, since our data is from the only tertiary centre in the country, the SMM rates observed here could be an overestimation of the true rate in the population.

Some limitations need to be highlighted. This is a retrospective study extracting data from documentation in medical records and, therefore, is limited by the variations and inaccuracies attributable to human error. The sample is restricted to one month of the year and is much smaller than previous studies- which could exaggerate the differences in the rates and performance of the index. The OBCMI needs further refinement since important risk factors such as ethnicity are not included. Furthermore, the index was generated and validated in different populations and clinical settings. Hence, although the index can be a valuable additional tool for risk assessment, further prospective studies exploring other risk factors and generating prediction models in the pregnant population of Qatar are required.

## Conclusion

The Obstetric comorbidity index has been validated worldwide and is an effective risk-assessment tool to identify high-risk pregnancies. The index performed moderately well in predicting SMM in pregnant women of Qatar and can be realistically used to red-flag pregnancies with multiple risk factors so that appropriate and timely multidisciplinary care can be initiated to reduce SMM and maternal mortality. To ensure consistent clinical practice, this study recommends using an OBCMI score ≥ 4 as the cut-off for assigning pregnancies with a high-risk status. The index is also helpful in predicting fetal morbidity; however, further prospective studies are required to validate OBCMI for CFM.

## APPENDIX 1: The OBCMI scoring system detailing the scores assigned to each comorbidity as extracted from reference 13 [13]

**Table:** 

Comorbidity	Score
Preeclampsia with severe features or eclampsia	5
Congestive heart failure	5
Pulmonary hypertension	4
Congenital heart and/or valvular disease	4
Placenta previa/ suspected accreta/ abruption	4
Ischemic heart disease/ Cardiac Arrhythmia	3
Sickle cell disease/ Bleeding disorder/ Coagulopathy/ Anticoagulation	3
Maternal age > 44 years	3
Body mass index > 50	3
Preeclampsia/Gestational/Chronic hypertension	2
Multiple gestation	2
Intrauterine fetal demise	2
Autoimmune disease/ Lupus	2
HIV/ AIDS	2
Epilespy/ cerebrovascular accident/ Neuromuscular disorder	2
Maternal age 40–44 years	2
Substance abuse	2
Body mass index > 40	2
Previous cesarean delivery/ myomectomy	1
Chronic renal disease	1
Asthma	1
Diabetic on insulin	1
Maternal age 35–39 years	1
Alcohol abuse	1

## Data Availability

The datasets generated and analysed during the current study are not publicly available due to hospital data-sharing policies and patient confidentiality but are available from the corresponding author upon reasonable request.

## Abbreviations

ACOG: American College of Obstetrics and Gynecology
APGAR: Appearance, Pulse, Grimace, Activity, Respiration
AUC: Area under curve
BMI: Body mass index
CFM: Cumulative fetal morbidity
CI: Confidence intervals
GA: Gestational age
HIE: Hypoxic ischemic encephalopathy
IQR: Interquartile range
IUFD: Intrauterine fetal death
NICU: Neonatal intensive care unit
NNH: Number needed to harm
NPV: Negative predictive value
OBCMI: Obstetrics Comorbidity Index
OR: Odds ratios
QEWS: Qatar early warning scores
RD: Risk difference
ROC: Receiver Operative Characteristic
SD: Standard deviation
SMM: Severe maternal morbidity
SROM: Spontaneous rupture of membranes
STROBE: Strengthening the reporting of Observational studies in Epidemiology
WWRC: Women’s Wellness and Research Centre
